# Genetic diversity of *Ophiocordyceps sinensis*, a medicinal fungus endemic to the Tibetan Plateau: Implications for its evolution and conservation

**DOI:** 10.1186/1471-2148-9-290

**Published:** 2009-12-16

**Authors:** Yongjie Zhang, Lingling Xu, Shu Zhang, Xingzhong Liu, Zhiqiang An, Mu Wang, Yinglan Guo

**Affiliations:** 1 Key Laboratory of Systematic Mycology and Lichenology, Institute of Microbiology, Chinese Academy of Sciences, No.3, 1st Beichen West Road, Chaoyang District, Beijing 100101, PR China; 2 School of Life Sciences, Shanxi University, Wucheng Road, Xiaodian District, Taiyuan, Shanxi 030006, PR China; 3 Graduate University, Chinese Academy of Sciences, Beijing 100049, PR China; 4 Epitomics, Inc., 863 Mitten Road, Suite 103, Burlingame, CA 94010-1303, USA; 5 School of Plant Science & Technology, Agriculture and Animal Husbandry College of Tibet, Nyingchi, Tibet 860000, PR China

## Abstract

**Background:**

*Ophiocordyceps sinensis *(syn. *Cordyceps sinensis*), endemic to alpine regions on the Tibetan plateau, is one of the most valuable medicinal fungi in the world. Huge commercial demand has led to excessive harvest and a dramatic decline in its numbers. The diversity of terrains and climates on the Tibetan Plateau and the broad insect host range (more than 50 species in the family Hepialidae) may have resulted in substantial intraspecific genetic diversity for this fungus. The objective of this study was to evaluate the population distribution of *O. sinensis *from geographically diverse regions of the Tibetan Plateau based on nrDNA ITS and *MAT1-2-1 *gene sequences. Understanding of the genetic diversity and genesis of *O. sinensis *will provide important information for the evolution and conservation of this fungus.

**Results:**

Significant sequence variations in the ITS and *MAT1-2-1 *genes (27 and 23 informative sites, eight and seven haplotypes, respectively) were observed. Phylogenetic analysis based on ITS sequences, *MAT1-2-1 *sequences, or their combined data set, clustered isolates from northern regions in one clade (clade I), whereas isolates from southern regions were dispersed in all four clades (clade I-IV). Single-strand conformation polymorphism (SSCP) analyses of 2639 ITS clones from seven samples revealed 91 different SSCP patterns that were subsequently sequenced. ITS heterogeneity was found in XZ-LZ07-H1 (Nyingchi population), and 17 informative sites and five haplotypes were detected from 15 clones. The five haplotypes clustered into three clades (clade I, II, and IV).

**Conclusions:**

Significant genetic divergence in *O. sinensis *was observed and the genetic diversification was greater among southern isolates than that among northern isolates. The polymorphism of nrDNA ITS sequences suggested that *O. sinensis *spread from a center of origin (the Nyingchi District) to southern regions and subsequently to northern areas. These results suggest that southern populations are important reservoirs of genetic diversity and should be taken into account in conservation programs.

## Background

The ascomycete *Ophiocordyceps sinensis *(Berk.) Sung, Sung, Hywel-Jones and Spatafora (syn. *Cordyceps sinensis*) [1], commonly known as the Chinese caterpillar fungus, has been widely used in traditional Chinese medicine for the treatment of asthma, bronchial, lung inflammation, and other diseases [2]. The medicinal activity is from the complex of the moth caterpillar parasitized by the fungus and the fungal stroma biomass. The caterpillars, which belong to the family Hepialidae, live underground in soil burrows in the Tibetan plateau and feed on plant roots. When an infection is established, the fungus grows within body cavity of the insect larvae and eventually kills and mummifies them in the underground burrows. Fungal fruiting body emerges from the front end of the caterpillar and pokes out of the ground in spring and early summer [3].

The natural distribution of this fungus, however, is limited to alpine regions on the Tibetan Plateau and huge commercial demand has led to excessive harvest and a dramatic decline in its numbers [4]. As a result, the price for natural *O. sinensis *has increased rapidly and it cost as much as $32,000 (USD) per kg for top quality in late 2006 [5]. Although several fermented mycelial products from the anamorph (*Hirsutella sinensis*) of the fungus have been commercialized in China [6], the teleomorph, which is the component for traditional Chinese medicine, has not yet been commercially cultivated. The natural population of this fungus needs to be preserved for the fragile Tibetan Plateau ecosystem and for sustainable supply of this natural resource.

*Ophiocordyceps sinensis *occupies a diverse habitat on the Tibetan Plateau, which is the largest and highest plateau on Earth, covering more than 2.5 million km^2 ^at an average elevation of over 4.5 km [7]. It is surrounded by and interspersed with towering mountain ranges. Its complex geomorphology and climate generate substantial interregional variation which results in remarkable faunal and floral diversity and high levels of endemism [8-11]. *O. sinensis *is endemic to the Tibetan Plateau and occurs at elevations ranging from 3000 m up to the snow line [4]. This distribution pattern suggests that the evolution of *O. sinensis *was significantly influenced by the tectonic movements during the strong uplift of the Tibetan Plateau. The spread of *O. sinensis*, depending on the shooting of ascospores, is limited to defined areas in a mountain and is unlikely through discontinuous mountains. Therefore, gene flow between different populations on discontinuous mountains is expected to be low, and divergence may have occurred [12].

Intraspecific genetic diversity of *O. sinensis *may also be generated by its wide host range. *O. sinensis *infects soil-borne larvae of more than 50 species of ghost moths, mostly in the genus *Hepialus *and to a lesser extent in the genera *Hepialiscus*, *Forkalus*, and *Bipectilus *[13,14]. Coevolution between the fungus and its host insects may have originated a long time ago [15]. Although the host insects in general have a limited distribution in the Tibetan Plateau and their distribution varies among different mountain ranges and even from different sides and habitats of the same mountain [16], the greater mobility of moths may also drive the genetic diversity of the fungus.

To date, genetic diversity of *O. sinensis *has only been studied at the local level of limited geographic areas. Three populations found in the north (Menyuan, Maqu, and Luqu), the middle (Yushu and Chengduo), and the south (Baima Snow Mountain, Renzhi Snow Mountain, Dacaodi, and Chongcaoxiawa) of the Tibetan Plateau, have been recognized from 29 samples based on RAPD analyses [12]. Inter-simple sequence repeat (ISSR) analyses of *O. sinensis *from 11 counties in Qinghai Province also resulted in three groups (around the Qinghai Lake, central eastern Qinghai, and southern Qinghai) [17]. Based on ITS sequences of samples from 11 localities in southwestern China, *O. sinensis *was divided into two populations (one from southwestern Sichuan to the northern slopes of the Himalayan Mountains, the other from northern Sichuan to Qinghai and Gansu) [18]. However, ITS sequence analyses in another study did not indicate any subgroup for 17 *O. sinensis *samples from different geographical regions [19]. Overall, the number of *O. sinensis *samples used in earlier studies was limited, and more samples from Tibet, a major production area, should be surveyed. In addition, other molecular markers, especially protein-encoding genes, should be employed to clarify the genetic diversity and to refine the phylogenetic origin of this fungus.

Mating-type genes play an important role in the evolution of fungal species. Sexual development is controlled by a single mating-type locus (*MAT*) in the fungi of Ascomycota. The term "idiomorph" instead of "allele" is usually used to describe the two alternate forms (*MAT1-1 *and *MAT1-2*) at the mating-type locus [20]. Mating-type genes evolve at a faster rate than other sequences, such as ITS and glyceraldehyde 3-phosphate dehydrogenase [21]. Although *MAT1-2-1 *sequences have been used to investigate the phylogenetic relationships among closely related species and usually provide high resolution [22], other studies found that the variability of *MAT1-2-1 *within species is low [21]. A putative mating-type gene (*MAT1-2-1*) of *O. sinensis *was cloned in our laboratory. Similar to *MAT1-2-1 *orthologs of other ascomycetes, the *O. sinensis MAT1-2-1 *gene contained the conserved HMG motif. The sequence homologies to *Cordyceps militaris *and *Cordyceps takaomontana *were ~50% between cDNA sequences and ~40% between amino acid sequences (Zhang *et al*., unpublished data). During our preliminary experiment, several distinctive base changes within the *MAT1-2-1 *sequences were found among some *O. sinensis *isolates. Therefore, this gene was selected to investigate whether it can provide reasonable information on the genetic diversity of *O. sinensis*.

The objective of this study was to evaluate the population distribution of *O. sinensis *from geographically diverse regions of the Tibetan Plateau based on nrDNA ITS and *MAT1-2-1 *gene sequences. Understanding of the genetic diversity and genesis of *O. sinensis *should provide valuable information for the protection and sustainable utilization of this natural resource.

## Results

### Sequence variations of nrDNA ITS and *MAT1-2-1* genes among *O. sinensis* isolates

Fifty-six *O. sinensis *isolates from different geographical regions were used in this study (Additional file 1). Their nrDNA ITS and *MAT1-2-1 *sequences were analyzed. For the 531-535 bp ITS sequences, the detectable nucleotide variations occurred at 35 sites, including 27 parsimony informative sites and eight singleton variable sites. Among the 27 parsimony informative sites, 13 were T/C base transitions; eight were A/G transitions; five were transversions of T/G, T/A, or A/C; and one was T/A/C substitution (see Additional file 2). Most of these informative sites were located in the ITS1 (11 sites) and ITS2 regions (14 sites), plus one each in the 5.8S and 28S regions (at sites 319 and 514, respectively). The singleton sites were not included in the subsequent haplotype analysis because one polymorphism at such a site was represented by only one isolate and such variation might be introduced artificially during PCR or sequencing steps. In addition, a 4-bp deletion was detected within the ITS2 region of two isolates (XZ-LZ07-H1 and XZ-LZ07-H2).

Detectable nucleotide variations within the 877-882 bp *MAT1-2-1 *sequences occurred at 28 sites, including 23 parsimony informative sites and five singleton variable sites. There were 10 T/C transitions, seven A/G transitions, and six C/G transversions (see Additional file 3). Nucleotide variations in nine sites resulted in amino acid changes. Interestingly, the two isolates that had base deletions within the ITS region contained a 5-bp insertion within the first intron of the *MAT1-2-1 *gene.

### Haplotypes within the 56 *O. sinensis *isolates

**Figure 1:**
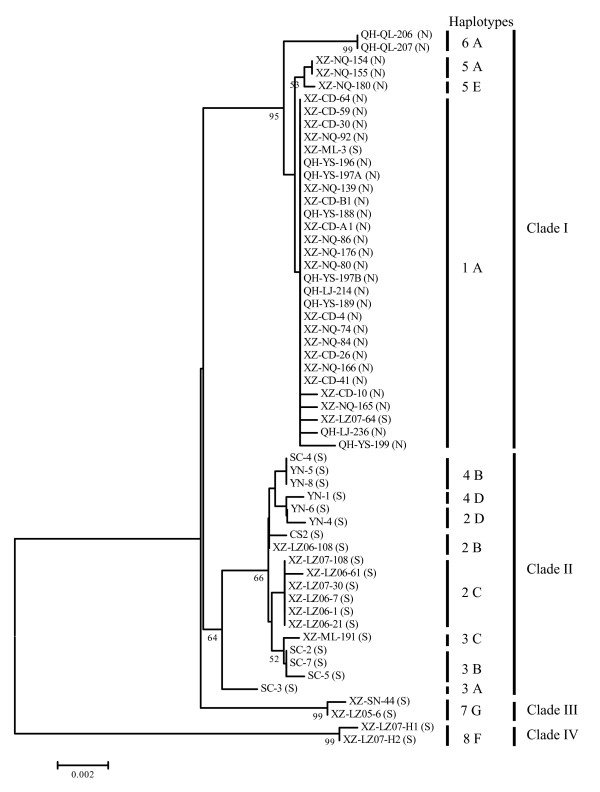
**Phylogenetic analyses of 56 *O. sinensis *isolates based on the combined data set of ITS and *MAT1-2-1***. Bootstrap values lower than 50% are not shown (the same hereinafter). Arabic numbers (1-8) indicate ITS haplotypes and capital letters (A-G) indicate *MAT1-2-1 *haplotypes. "N" or "S" behind the names of isolates represents northern or southern isolates, respectively.

Haplotype usually refers to a group of alleles at multiple loci that are transmitted together on a single chromosome or to a set of single nucleotide polymorphisms (SNPs) [23]. Ascosporic fungi are haploid and haplotypes were identified in this study according to base change patterns at informative sites among individual isolates. For the 56 isolates used in this study, eight ITS haplotypes, seven *MAT1-2-1 *haplotypes, and 14 combined haplotypes were detected (Figure 1, Additional file 1). The results showed that one haplotype might include isolates from different geographical regions (e.g., ITS type 7 and *MAT1-2-1 *type G), and isolates from the same location (e.g., XZ-NQ-176 and XZ-NQ-180) might contain different haplotypes.

For the eight ITS haplotypes (1-8), type 1 was the most dominant and it was represented by 28 isolates mainly from northern regions (Nagqu, Chamdo, Yushu, and Laji). Type 2 included nine isolates mainly from southern regions (Nyingchi and Baima). Other haplotypes were represented by two to five isolates (Figure 1, Additional file 1).

For the seven *MAT1-2-1 *haplotypes (A-G), type A was the most dominant and widespread; and it was represented by 33 isolates mainly from northern regions (Nagqu, Chamdo, Yushu, Laji, and Qilian). The next most common haplotype was type B, which included seven isolates from southern regions, such as Nyingchi, Baima, and Garze. Type C was represented by seven isolates from southern Tibet (Nyingchi and Mila). Other haplotypes were represented by one to three isolates (Figure 1, Additional file 1).

For the 14 combined haplotypes (Figure 1), type 1A was the most dominant and it included 28 isolates. The next most common combined haplotype was type 2C, which was represented by six isolates from Nyingchi. Other haplotypes were represented by one to three isolates.

### Phylogenetic analyses

Minimum evolutionary phylogenetic analyses were performed with sequences of the nrDNA ITS region, the *MAT1-2-1 *gene, and their combined data set. In these phylogenetic trees, two major clades (I and II) and two small clades (III and IV) were distinguished (Figure 1, see also Additional file 4).

Clade I (north clade) was composed of isolates mainly from northern Tibet (Nagqu and Chamdo) and Qinghai (Yushu, Laji, and Qilian) with the exception of several isolates from southern regions (XZ-ML-3, XZ-LZ07-64, and/or SC-3). This clade received bootstrap support values of 86%, 73%, and 95% in three phylogenetic trees, and included three ITS haplotypes, two *MAT1-2-1 *haplotypes, and four combined haplotypes (Figure 1, Additional file 4).

Clade II was composed of southern isolates from southern Tibet (Nyingchi and Mila), Yunnan, and Sichuan. It had bootstrap support of 91%, 60%, and 64% in the three phylogenetic trees, and included three ITS haplotypes, three *MAT1-2-1 *haplotypes, and eight combined haplotypes (Figure 1, Additional file 4).

Two southern isolates, XZ-SN-44 and XZ-LZ05-6, clustered together as the third clade (clade III), having 97%, 99%, and 99% bootstrap support in the three trees (Figure 1, Additional file 4). The southern isolates XZ-LZ07-H1 and XZ-LZ07-H2 formed the fourth clade (clade IV) and had a distant relationship with other isolates (Figure 1, Additional file 4). Among all tested isolates, the maximal ITS distance value was 0.043 between XZ-LZ07-H1 and QH-QL-206 or QH-QL-207. The maximal *MAT1-2-1 *distance value was 0.023 between YN-4 and XZ-LZ07-H1 or XZ-LZ07-H2.

The phylogenetic trees constructed with ITS sequences and with *MAT1-2-1 *sequences had very similar topological structures (see Additional file 4). Four clades were discriminated in both trees, and composition of isolates for each clade was almost identical between the two trees. However, there were some discrepancies between the two trees. For example, isolate SC-3 clustered in clade II in the ITS tree, but in clade I in the *MAT1-2-1 *tree. Clade II was the sister group of clade III in the ITS tree, but the sister group of clade I in the *MAT1-2-1 *tree. The parameters of nucleotide diversity and haplotype diversity were usually higher in the ITS sequences than in the *MAT1-2-1 *sequences (Table 1). Therefore, the ITS sequence is relatively more polymorphic than the *MAT1-2-1 *sequence.

**Table 1:** Comparison of genetic diversity parameters^a^

Populations^b^	No. of sequences	No. of polymorphic sites^c^	Average no. of nucleotide differences, *K*	Nucleotide diversity, *Pi *(×10^-2^)	Haplotype diversity, *h*
Qilian (N)	2	0 (0)	0.00 (0.00)	0.00 (0.00)	0.00 (0.00)
Laji (N)	2	1 (0)	1.00 (0.00)	0.19 ± 0.09 (0.00)	1.00 ± 0.50 (0.00)
Yushu (N)	6	1 (1)	0.33 (0.33)	0.06 ± 0.04 (0.04 ± 0.02)	0.33 ± 0.22 (0.33 ± 0.22)
Nagqu (N)	12	2 (1)	0.58 (0.17)	0.11 ± 0.03 (0.02 ± 0.01)	0.53 ± 0.14 (0.16 ± 0.13)
Chamdo (N)	9	1 (0)	0.22 (0.00)	0.04 ± 0.03 (0.00)	0.22 ± 0.17 (0.00)
Nyingchi (S)	11	22 (23)	6.51 (7.42)	1.25 ± 0.44 (0.85 ± 0.27)	0.62 ± 0.16 (0.80 ± 0.11)
Mila (S)	2	7 (4)	7.00 (4.00)	1.31 ± 0.66 (0.46 ± 0.23)	1.00 ± 0.50 (1.00 ± 0.50)
Garze (S)	5	2 (5)	0.80 (2.00)	0.15 ± 0.09 (0.23 ± 0.11)	0.40 ± 0.24 (0.70 ± 0.22)
Baima (S)	5	1 (2)	0.60 (1.00)	0.11 ± 0.03 (0.11 ± 0.03)	0.60 ± 0.18 (0.80 ± 0.16)
Northern regions	31	10 (2)	1.06 (0.13)	0.20 ± 0.07(0.02 ± 0.01)	0.49 ± 0.11 (0.13 ± 0.08)
Southern regions	25	26 (27)	4.37 (5.01)	0.82 ± 0.26 (0.57 ± 0.17)	0.83 ± 0.06 (0.87 ± 0.04)
Overall	56	35 (28)	4.45 (3.35)	0.84 ± 0.13 (0.38 ± 0.09)	0.78 ± 0.05 (0.67 ± 0.06)

^a ^Fifty-six individuals were sequenced in this study (Additional file 1). "N" or "S" inside parentheses represents northern or southern populations, respectively (the same hereinafter). Numbers outside parentheses are values of ITS, and numbers inside parentheses are values of *MAT1-2-1 *(the same hereinafter). ^b ^Genetic parameters of the Shannan population and the unknown population (Additional file 1) are included in the "southern regions" and "overall" statistics, but they are not given individually because they each possess one isolate.^c ^The "polymorphic sites" include both informative sites and singleton sites. For both ITS and *MAT1-2-1*, there is one polymorphic site shared by northern and southern isolates.

### Population structure and gene flow

For both ITS and *MAT1-2-1*, significant genetic differentiation was detected among isolates in Nyingchi versus Yushu, Nagqu, Chamdo, and Garze; Garze versus Nagqu, Chamdo, and Nyingchi; and Baima versus Yushu, Nagqu, and Chamdo (Table 2). In addition, there was also significant differentiation for ITS sequences among isolates in Nagqu versus Qilian; Nyingchi versus Qilian; and Garze versus Yushu (Table 2). Analyses of molecular variance (AMOVA) based on haplotype frequencies showed significant *F*_CT _values in the following population grouping patterns: [south isolates] [north isolates]; [Nyingchi] [other south isolates] [north isolates]; [Garze] [other south isolates] [north isolates]; and [Garze] [other south isolates] [Qilian and Laji] [other north isolates] (Table 3). Significant difference (*P *< 0.01) was observed for these grouping patterns. These results indicate that an organized population structure exists among *O. sinensis *isolates.

**Table 2:** Pairwise population differences and *F*_ST _values for ITS and *MAT1-2-1 *sequences

	Qilian (N)	Laji (N)	Yushu (N)	Nagqu (N)	Chamdo (N)	Nyingchi (S)	Mila (S)	Garze (S)	Baima (S)
Qilian (N)	0.00 (0.00)	0.50 (0.00)	0.73 (-0.30)	0.56** (-0.33)	0.81 (0.00)	0.49** (0.31)	0.50 (0.00)	0.69 (0.48)	0.55 (0.42)
Laji (N)	0.50 (0.00)	1.00 (0.00)	0.09 (-0.30)	0.01 (-0.33)	0.27 (0.00)	0.29 (0.31)	-0.33 (0.00)	0.44 (0.48)	0.28 (0.42)
Yushu (N)	0.83 (0.00)	-0.08 (0.00)	0.33 (0.33)	0.01 (-0.03)	-0.06 (0.07)	0.49** (0.34**)	0.09 (0.09)	0.64** (0.50)	0.54** (0.45**)
Nagqu (N)	0.73 (0.00)	-0.10 (0.00)	0.01 (-0.01)	0.53 (0.17)	0.06 (-0.02)	0.43** (0.48**)	0.01 (0.38)	0.52** (0.66**)	0.44** (0.62**)
Chamdo (N)	0.89 (0.00)	-0.06 (0.00)	-0.02 (0.00)	0.03 (0.00)	0.22 (0.00)	0.56** (0.53**)	0.27 (0.66)	0.71** (0.75**)	0.64** (0.71**)
Nyingchi (S)	0.69 (0.51)	0.19 (0.51)	0.52 (0.36)	0.43 (0.43)	0.58 (0.51)	0.62 (0.80)	0.29 (-0.19)	0.46** (0.20**)	0.18 (0.17)
Mila (S)	0.50 (0.00)	-0.25 (0.00)	-0.08 (-0.08)	-0.10 (-0.04)	-0.06 (0.00)	0.19 (-0.17)	1.00 (1.00)	0.02 (0.21)	0.28 (0.14)
Garze (S)	0.80 (0.65)	0.30 (0.65)	0.63 (0.48)	0.53 (0.57)	0.69 (0.65)	0.49 (0.20)	-0.10 (0.15)	0.40 (0.70)	0.43 (0.01)
Baima (S)	0.70 (0.60)	0.20 (0.60)	0.53 (0.43)	0.43 (0.52)	0.59 (0.60)	0.14 (0.16)	0.20 (0.10)	0.38 (0.01)	0.60 (0.80)

Notes: Above diagonal: pairwise *F*_ST _values between populations. Diagonal elements: average number of pairwise differences within populations (PiX). Below diagonal: corrected average pairwise difference (PiXY-(PiX+PiY)/2). Pairwise *F*_ST _values that are statistically different are indicated (*P *< 0.01).

**Table 3:** AMOVA for grouping of populations estimated using *F*-statistics based on ITS and *MAT1-2-1 *sequences

Groups	Among pops within groups (*F*_SC_)	Within pops (*F*_ST_)	Among groups (*F*_CT_)	Among groups (%)	Among groups (*p*)
[Nyingchi, Mila, Baima, Garze] [Qilian, Laji, Yushu, Nagqu, Chamdo]	0.10 (0.02)	0.24 (0.21)	0.14* (0.21**)	28.60 (47.73)	0.03 (<0.01)
[Nyingchi] [Mila, Baima, Garze, Qilian, Laji, Yushu, Nagqu, Chamdo]	0.15 (0.11)	0.24 (0.21)	0.07 (0.08)	15.82 (19.24)	0.32 (0.32)
[Nyingchi] [Mila, Baima, Garze] [Qilian, Laji, Yushu, Nagqu, Chamdo]	0.08 (0.00)	0.24 (0.21)	0.14* (0.20**)	30.55 (49.85)	0.02 (<0.01)
[Nyingchi, Mila, Baima] [Garze] [Qilian, Laji, Yushu, Nagqu, Chamdo]	0.07 (0.00)	0.24 (0.21)	0.16** (0.21**)	33.62 (49.71)	<0.01 (<0.01)
[Nyingchi, Mila, Baima] [Garze] [Qilian, Laji] [Yushu, Nagqu, Chamdo]	0.05 (0.01)	0.24 (0.21)	0.17** (0.17**)	36.43 (44.40)	<0.01 (<0.01)

Notes: *F*_SC _estimates the variation among populations relative to a regional grouping of populations. *F*_ST _estimates the proportions of genetic variation within populations relative to the genetic variation for the whole samples. *F*_CT _estimates the proportion of genetic variation among groups of populations relative to the whole species. *F*_CT _values that are statistically different are indicted. * *P *< 0.05; ** *P *< 0.01.

### ITS heterogeneity by PCR-SSCP analyses

**Figure 2:**
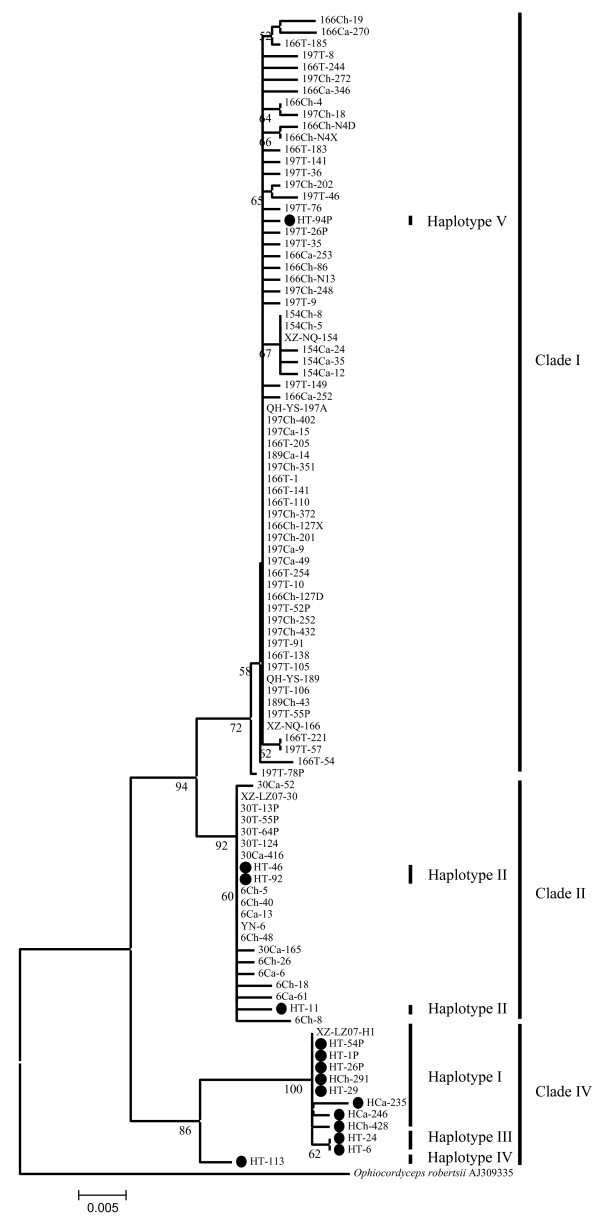
**Phylogenetic analyses based on ITS sequences of 91 clones and their original sequences from direct sequencing**. "•" indicates clones of XZ-LZ07-H1. Names of clones beginning with 154, 166, 189 and 197 originate from northern isolates XZ-NQ-154, XZ-NQ-166, QH-YS-189, and QH-YS-197B, respectively. Names of clones beginning with 6, 30, and H originate from southern isolates YN-6, XZ-LZ07-30, and XZ-LZ07-H1, respectively. Ca, Ch, and T indicate the source of clones from stromata, sclerotia, and external mycelial vela, respectively.

In total, 2639 ITS clones (1483 clones from four northern samples and 1156 clones from three southern samples) were analyzed by SSCP. Ninety-one clones with different SSCP patterns (60 for the northern samples and 31 for the southern samples) were sequenced (Table 4). Compared with the corresponding original sequence from direct sequencing, no ITS heterogeneity was detected for clones from each of these specimens except for XZ-NQ-166 and XZ-LZ07-H1, which had three (23 clones) and 17 informative sites (15 clones), respectively (Table 4). High nucleotide diversity (1.66 ± 0.34) and haplotype diversity (0.88 ± 0.07) were observed among clones of XZ-LZ07-H1. The 4-bp sequence deletion in XZ-LZ07-H1 from direct sequencing existed within some clones of XZ-LZ07-H1 (see Additional file 5). Among these 17 informative sites from 15 clones of XZ-LZ07-H1, 15 changes occurred at known informative sites and one at known singleton sites (see footnotes of Additional file 5). Every possible nucleotide change at these informative sites was shared by at least two to five clones. According to base changes at these informative sites, clones of XZ-LZ07-H1 can be divided into five haplotypes (I-V). The five haplotypes contained eight, three, two, one, and one clone(s), respectively (Figure 2). Clones from the four northern isolates were clustered together in agreement with their original sequences from direct sequencing (Figure 2). Similarly, clones from two of the three southern samples (XZ-LZ07-30 and YN-6) were clustered together correspondingly with their original sequences from direct sequencing (Figure 2). However, the 15 clones from isolate XZ-LZ07-H1 were scattered among three clades (clade I, II, and IV). It is noteworthy that HT-94P clustered with the northern isolates (clade I).

**Table 4:** PCR-SSCP results of four northern samples and three southern samples^a^

Specimens	No. of tested clones				No. of sequenced clones				No. of polymorphic sites^b^	Nucleotide diversity, *Pi*(×10^-2^)
				
	Stromata	Sclerotia	Mycelial vela	Total	Stromata	Sclerotia	Mycelial vela	Total		
XZ-NQ-166 (N)	285	247	145	677	4	8	11	23	3 (17)	0.37 ± 0.07
QH-YS-197B (N)	228	275	189	692	3	10	17	30	0 (18)	0.24 ± 0.05
QH-YS-189 (N)	36	16	-	52	1	1	-	2	0 (0)	0.00
XZ-NQ-154 (N)	40	22	-	62	3	2	-	5	0 (3)	0.23 ± 0.06
XZ-LZ07-30 (S)	255	234	16	505	3	0	4	7	0 (2)	0.11 ± 0.05
XZ-LZ07-H1 (S)	258	260	44	562	2	2	11	15	17 (10)	1.66 ± 0.34
YN-6 (S)	38	51	-	89	3	6	-	9	0 (9)	0.38 ± 0.11

Total	1140	1105	394	2639	19	29	43	91	26 (49)	1.24 ± 0.15

^a ^Two or three sections (stromata, sclerotia and/or external mycelial vela) were tested for each specimen.

^b ^Numbers outside parentheses are parsimony informative sites, inside parentheses singleton variable sites.

## Discussion

The distance value (*K *value) of nrDNA ITS sequences from one fungal species typically ranges from 0 to 0.05 [24,25]. The maximal distance value among *O. sinensis *populations in this study was 0.043. Although five putative new species related to *O. sinensis *were reported based on morphological characters and geographical distribution [26-28], they should not be treated as different species based on nrDNA ITS sequences [29]. A high degree of intraspecific genetic diversity of *O. sinensis *has been detected, but it was still highly dissimilar even from the most closely related lineages within same genus, having about 10% of ITS difference with *O. robertsii *(see Additional file 6).

In general, mating-type genes are more variable between species than within species [21] and the high degree of variations between species has been used to study the phylogeny of some closely related fungal groups [22]. In contrast to the low within-species variation (0.15-0.31% nucleotide sites different) of *Cochliobolus heterostrophus *[21] and *Phaeosphaeria nodorum *[30], our comparison of 56 *O. sinensis *isolates detected at least 23 base changes within 877-882 nucleotides of the *MAT1-2-1 *sequences (2.6%), including nine nonsynonymous variations. This suggests that the unique geophysical environment and wide insect host range have resulted in a high degree of intraspecific genetic diversity within *O. sinensis*. Mating-type genes are master regulators of the signal transduction pathway of mating [21], but whether variations within *MAT1-2-1 *sequence affect the sexual reproduction of *O. sinensis *is unknown. The fact that all strains contain the *MAT1-2-1 *gene suggests that it might be homothallic, capable of self-fruiting. However, more study is needed to clarify the mating system of *O. sinensis*. This study is the first attempt to use a mating-type gene to determine whether the gene sequence differs among *O. sinensis *isolates from different localities. This is also the first protein-encoding gene used for the genetic analyses of *O. sinensis *isolates from different regions.

**Figure 3:**
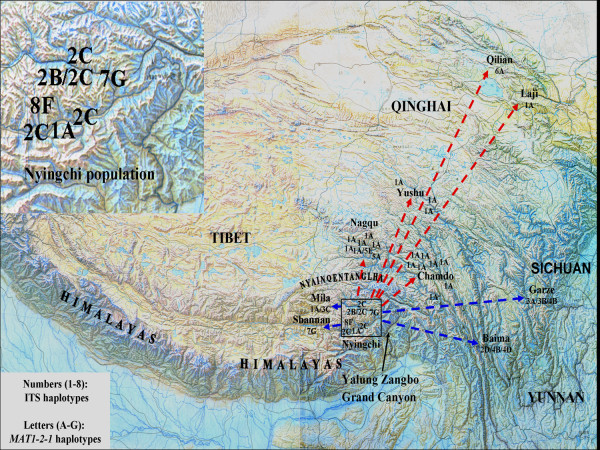
**Proposed center of origin and transmission pathway of *O. sinensis* on the Tibetan Plateau**. The figure at the upper left corner is the enlargement of Nyingchi District (proposed center of origin). Haplotypes of *O. sinensis* isolates at each sampling site are shown on the map. Original map was scanned from Liao, 1990 [41].

In this study, significant genetic divergence within the species of *O. sinensis *was revealed by two genes (nrDNA ITS and *MAT1-2-1*) and two main populations were detected: the southern population (Nyingchi, Mila, Shannan, Garze, and Baima) and the northern populations (Yushu, Qilian, Laji, Chamdo, and Nagqu). Diversification among southern isolates was greater than that among northern isolates. In addition, significant genetic differentiation was observed between Nyingchi and other populations. Nyingchi has greater within-population genetic diversity than other populations. ITS heterogeneity was detected in isolate XZ-LZ07-H1 from Nyingchi. These results imply that Nyingchi might have acted as a center of origin of *O. sinensis *and *O. sinensis *might have been transmitted from Nyingchi to other locations (Figure 3). *O. sinensis *might have spread eastward and westward first among southern regions on the Tibetan Plateau. This spread in southern Tibetan Plateau may be initiated along two large and parallel mountain ranges, the Himalaya Mountains (south of Nyingchi) and the Nyainqentanglha Mountains (north of Nyingchi), which run in east and west directions. The spread from south to north on the Tibetan Plateau might have occurred at a later time and this may be achieved by air currents from the Indian Ocean through Yarlung Zangbo Grand Canyon.

Both *O. sinensis *and its host insects are endemic species on the Tibetan Plateau; and the geography and climate may have played an important role in their evolution and coevolution. However, the broad range of host insects and their distribution characters may also have impacted on the diversification of *O. sinensis *because the fungus must parasitize caterpillar to complete its life cycle [13,14,16]. Host species of the fungal isolates used in this study and the genetic diversity of the hosts were not determined. The whole picture of *O. sinensis *genetic differentiation and the coevolution between *O. sinensis *and host insects needs to be further studied [15].

Two southern isolates, XZ-LZ07-H1 and XZ-LZ07-H2, had relatively distant genetic relationships from other isolates. Compared to the ITS or *MAT1-2-1 *sequences of other isolates, the sequences of these two isolates had two fixed 4- or 5-bp indels and 13 unique base changes. According to the newly proposed "indel-associated substitution" theory, the high substitution frequency in these two isolates may be related to the existence of the two indels. The theory asserts that the single-nucleotide mutation rate increases in regions surrounding the insertion or deletion sites [31]. Furthermore, ITS heterogeneity was detected in XZ-LZ07-H1, but the potential sources of heterogeneity are still unknown. The fact that one clone of XZ-LZ07-H1 clustered in the northern clade suggests that hybridization is one of the possible mechanisms. Inherent mechanisms involving slippage events during DNA duplication may also be one of the reasons for the existence of base indels in the sequence of XZ-LZ07-H1 and XZ-LZ07-H2.

The considerable base changes detected in this study indicated that recombination might have occurred between different *O. sinensis *populations. Considering the difficulty of gene flow among different populations, *O. sinensis *may have an ancestral origin. According to a recent report, although diversification of insect pathogens within the fungal order of Hypocreales occurred during the Cretaceous period, the genesis of *O. sinensis *was an event during the Cenozoic era (65.5 million years ago (Mya) to present) [32]. We suggest the history of *O. sinensis *was not earlier than 1.8 Mya, because the altitude of the Tibetan Plateau reached ~2000 m in the Eupleistocene (1.8 to 0.8 Mya) and it has kept a trend of rapid uplifting since then [33].

## Conclusions

Based on two DNA sequences (nrDNA ITS and *MAT1-2-1*), significant genetic divergence was detected among 56 isolates of *O. sinensis *collected from different regions of the Tibetan Plateau. The genetic diversification was greater among southern isolates than that among northern isolates, indicating that southern populations were important genetic reservoirs of this species to be considered in conservation programs. The polymorphism of nrDNA ITS sequences suggests that the Nyingchi District is the center of origin of *O. sinensis*; and that the fungus may have first spread from this center first to other southern regions and then to northern areas.

## Methods

### *Ophiocordyceps sinensis*

Fifty-six natural specimens representing 11 populations of *O. sinensis *were collected from the Tibet Autonomous Region, Qinghai Province, Sichuan Province, and Yunnan Province in southwestern China during 2005 and 2008 (Additional file 1). After dug out from the soil, each specimen was placed into a new zip plastic bag immediately. They were taken back to our laboratory by keeping at low temperature in a portable refrigerator and stored at 4°C after arrival. Anamorphic strains were isolated from stromatal tissues within three weeks according to the protocol of Liu *et al *[34]. The remaining fruiting bodies were stored at -20°C. Either axenic isolates or the stromatal section of natural specimens were used for DNA analysis.

### Amplification of sequences from the nrDNA ITS region

Genomic DNA was extracted with the CTAB method [35]. ITS regions of nrDNA were amplified with the primers ITS5 (5'-GGAAGTAAAAGTCGTAACAAGG-3') and ITS4 (5'-TCCTCCGCTTATTGATATGC-3') using high-fidelity *Pfu *DNA polymerase (TIANGEN BIOTECH, China). The PCR mixture contained 5 μl of 10 × Pfu buffer, 5 μl of dNTP mixture (2.5 mM), 2 μl of each primer (10 μM), 2 μl of deionized formamide, 1 μl of MgCl_2 _(25 mM), 1 μl of genomic DNA, and 0.5 μl of *Pfu *DNA polymerase in a total volume of 50 μl. PCR amplification was performed in a TG_RADIENT _thermocycler (Biometra, Germany). The mixture was heated for 5 min at 94°C; then subjected to 35 cycles of 40 s at 94°C, 40 s at 54°C, and 60 s at 72°C; and a final 10 min elongation step at 72°C. After confirmation of the PCR products by agarose gel electrophoresis, the fragments were cleaned using the 3S Spin PCR Product Purification Kit (Biocolor Bioscience & Technology Company, China). The purified PCR products were then sequenced with the primer ITS5 using an ABI 3730 XL DNA sequencer with BigDye 3.1 Terminators (Applied Biosystems, USA).

### Amplification of sequences for *MAT1-2-1* gene

The *MAT1-2-1 *gene of *O. sinensis *was first cloned in our laboratory using degenerate primers, and both flanks were extended using DNA Walking *SpeedUp*™ Premix Kit (Seegene, Korea) (Zhang *et al*., unpublished data). For this study, *MAT1-2-1 *sequences were amplified with the primers MAT1-2F1 (5'-CCACCGATCCAAGTCTCCT-3') and MAT1-2R2 (5'-CAGTTTCAGTCGCTGTCGTG-3'). The assay included: 5 μl of 10× Pfu buffer, 5 μl of dNTP mixture (2.5 mM), 2 μl of each primer (10 μM), 1 μl of genomic DNA, 0.5 μl of *Pfu *DNA polymerase, and water to a final volume of 50 μl. The PCR profile consisted of: 94°C for 5 min; followed by 35 cycles of 94°C for 40 s, 55°C for 40 s, and 72°C for 70 s; with a final 10 min incubation at 72°C. PCR products were purified, and sequencing was done in both directions using both the forward and reverse primers following the protocol described above.

### SSCP analysis

SSCP is the electrophoretic separation of single-stranded nucleic acids based on subtle differences in sequence (often a single base pair). We used this technology to identify ITS heterogeneity of *O. sinensis*. Based on the results from analyses of ITS sequences, four samples from northern regions (QH-YS-197B, QH-YS-189, XZ-NQ-154, and XZ-NQ-166) and three samples from southern regions (XZ-LZ07-30, XZ-LZ07-H1, and YN-6) were selected for SSCP analyses. Each sample was divided into two or three sections (stromata, sclerotia, and/or external mycelial vela), and genomic DNA was extracted separately from each section. ITS sequences were amplified with the primers ITS1 (5'-TCCGTAGGTGAACCTGCGG-3') and ITS4 using the same conditions described for amplification of nrDNA ITS sequences. The exception was that *Taq *DNA polymerase (TaKaRa, Japan) instead of *Pfu *was used for the convenience of subsequent cloning. The PCR products were ligated into the TA plasmid pMD18-T (TaKaRa, Japan) and transformed into *Escherichia coli *DH5α. Colony PCR was then conducted to amplify the ITS1 region with the primers ITS1 and ITS2 (5'-GCTGCGTTCTTCATCGATGC-3'). Clones with expected amplicons were used for SSCP analyses following the procedure of Wang *et al *[36]. Different migration profiles between clones were compared using the Image J software. Representative clones of distinct patterns were sequenced with the universal primer M13-47.

### Sequence analyses

Sequences were aligned with Clustal X [37], and ambiguous regions in both sides were excluded from the subsequent analyses. For the ITS region, 531-535 bp of sequences including partial 18S (5 bp), ITS1 (160 bp), 5.8S (157 bp), ITS2 (171-175 bp), and partial 28S (38 bp) were used. For the *MAT1-2-1 *gene, 877-882 bp of sequences from 14 bases upstream of the start codon to six bases downstream of the stop codon were used. Pairwise distance matrices and minimum evolutionary (ME) phylogenetic analyses were conducted with the Kimura 2-Parameter model using MEGA 4 software [38]. To assess the confidence of phylogenetic relationships, the bootstrap tests were conducted with 1000 resamplings. *Ophiocordyceps robertsii *was used as the outgroup. DnaSP software (version 4.50.3) was used to estimate the genetic parameters of nucleotide diversity [39]. Genetic differentiation between populations and the analyses of molecular variance (AMOVA) were assessed using the program Arlequin 3.11 [40].

## Authors' contributions

YJZ, MW and YLG collected natural *O. sinensis *and isolated strains in the laboratory. YJZ and SZ carried out the PCR amplification of nrDNA ITS and *MAT1-2-1 *sequences, participated in the sequence alignment and drafted the manuscript. YJZ and MW conducted PCR-SSCP analysis. LLX and YLG participated in the bioinformatic analysis. XZL and ZQA conceived the study, participated in its design and coordination, and prepared the last version of the manuscript. All authors read and approved the final manuscript.

## Supplementary Material

Additional file 1 ***Ophiocordyceps sinensis *isolates used in this study**. ^a ^Populations were defined according to administrative divisions. For each population, natural *O. sinensis *were collected from one to eight different mountains and usually came from different towns or counties. "N" or "S" inside parentheses represents northern or southern populations, respectively.^b ^See Additional file 2 and 3 for detailed nucleotide variation among ITS haplotypes and *MAT1-2-1 *haplotypes, respectively. Click here for file

Additional file 2 **Sequence differences among eight haplotypes found in the nrDNA ITS region^a^**. ^a ^"NO" indicates one base deletion for haplotype 8 at position of 492. Click here for file

Additional file 3 Sequence differences among seven haplotypes found in the *MAT1-2-1 *sequences. Click here for file

Additional file 4 **Phylogenetic analyses of 56 *O. sinensis *isolates based on ITS sequences (A) and *MAT1-2-1 *sequences (B)**. Bootstrap values lower than 50% are not shown. Click here for file

Additional file 5 **Alignment of ITS sequences from direct sequencing and from clones of XZ-LZ07-H1**. Description: Dashes and dots indicate identity and indel, respectively. HCa, HCh, and HT represent clones from stromata, sclerotia, and external mycelial vela, respectively. From these clones, 17 informative sites were detected, of which 15 occurred at previously known informative sites (6, 7, 13, 25, 30, 115, 132, 350, 379, 395, 436, 442, 446, 463, 492), one at a previously known singleton site (32). Click here for file

Additional file 6 Similarity of ITS sequences for *O. sinensis *and other taxa * (%). Description: * These taxa were chosen according to Sung *et al *(2007). Click here for file
