# Supplementary figures and images for: Genetic diversity of Ophiocordyceps sinensis, a medicinal fungus endemic to the Tibetan Plateau: Implications for its evolution and conservation
Source: BMC Evol Biol. 2009 Dec 16;9:290. doi: 10.1186/1471-2148-9-290 (PMC2805636; doi:10.1186/1471-2148-9-290)

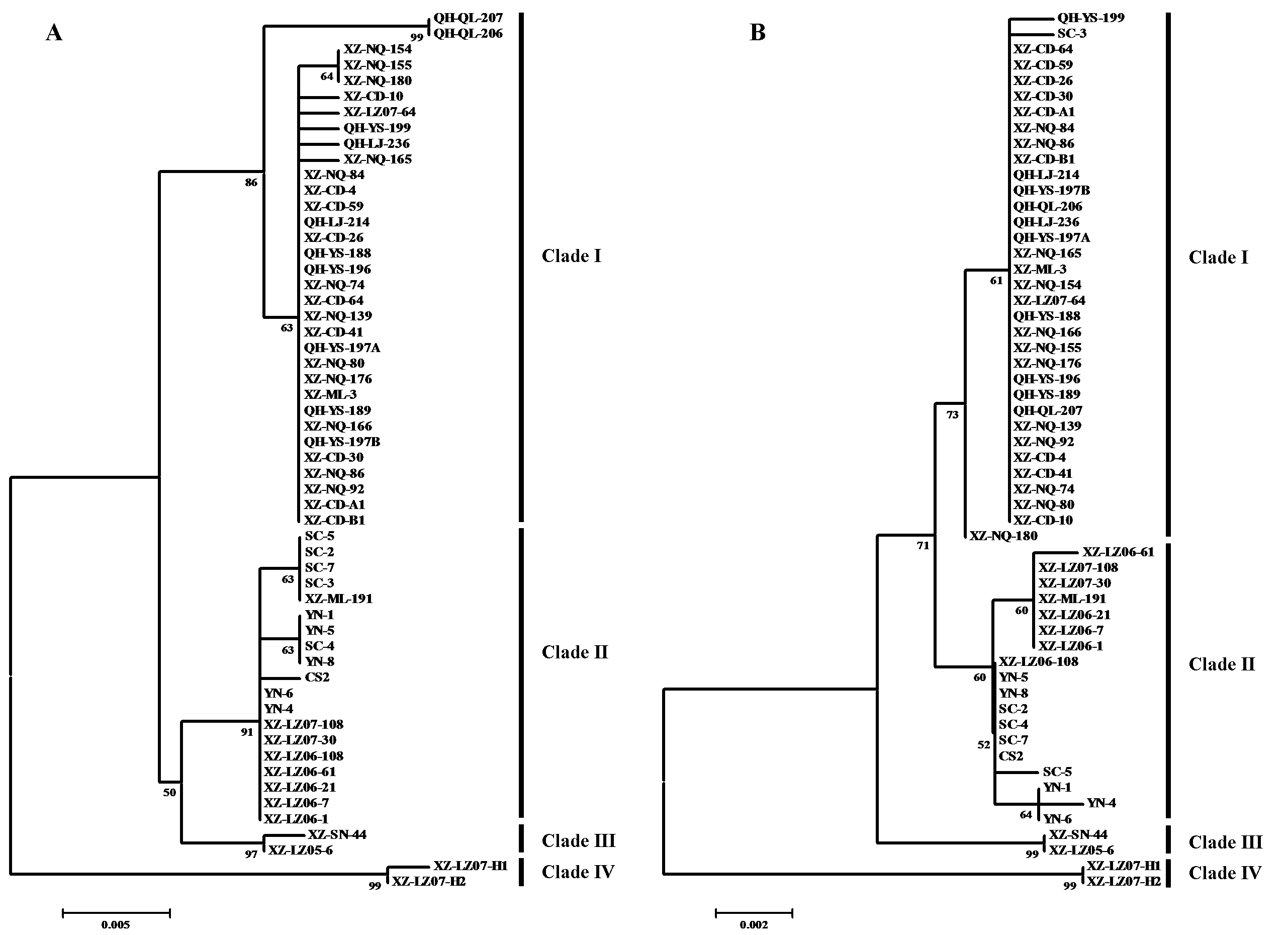

Supplement: Additional file 4 — Phylogenetic analyses of 56 O. sinensis isolates based on ITS sequences (A) and MAT1-2-1 sequences (B). Bootstrap values lower than 50% are not shown. [file 1471-2148-9-290-S4.TIFF]

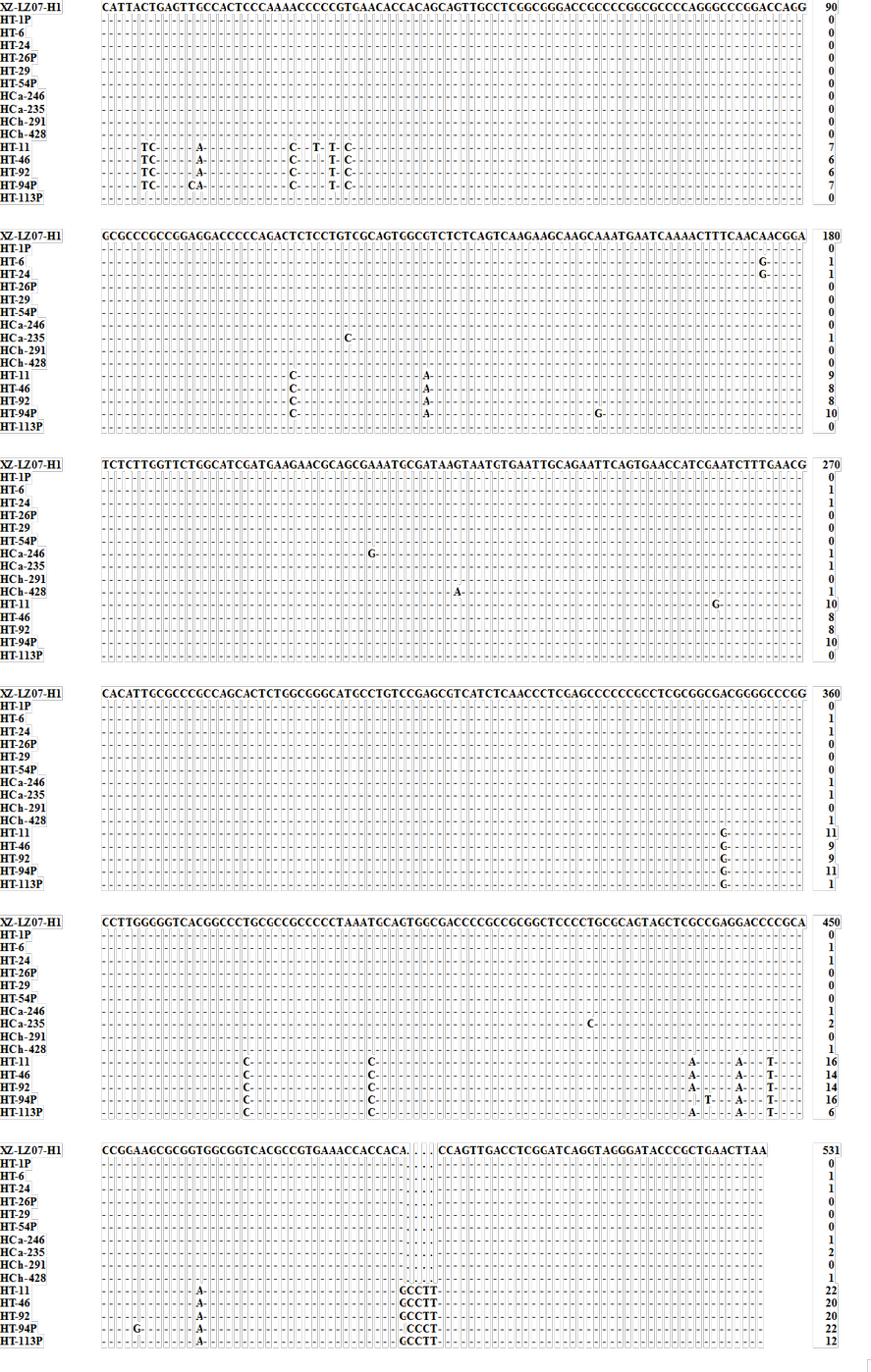

Supplement: Additional file 5 — Alignment of ITS sequences from direct sequencing and from clones of XZ-LZ07-H1. Description: Dashes and dots indicate identity and indel, respectively. HCa, HCh, and HT represent clones from stromata, sclerotia, and external mycelial vela, respectively. From these clones, 17 informative sites were detected, of which 15 occurred at previously known informative sites (6, 7, 13, 25, 30, 115, 132, 350, 379, 395, 436, 442, 446, 463, 492), one at a previously known singleton site (32). [file 1471-2148-9-290-S5.TIFF]
